# Dysbiosis May Trigger Autoimmune Diseases via Inappropriate Post-Translational Modification of Host Proteins

**DOI:** 10.3389/fmicb.2016.00084

**Published:** 2016-02-05

**Authors:** Aaron Lerner, Rustam Aminov, Torsten Matthias

**Affiliations:** ^1^The Ruth and Bruce Rappaport Faculty of Medicine, Technion-Israel Institute of TechnologyHaifa, Israel; ^2^AESKU.KIPP InstituteWendelsheim, Germany; ^3^School of Medicine and Dentistry, University of AberdeenAberdeen, UK

**Keywords:** microbiome, dysbiosis, intestine, post-translational modification, autoimmune disease

## Abstract

The gut ecosystem with myriads of microorganisms and the high concentration of immune system cells can be considered as a separate organ on its own. The balanced interaction between the host and microbial cells has been shaped during the long co-evolutionary process. In dysbiotic conditions, however, this balance is compromised and results in abnormal interaction between the host and microbiota. It is hypothesize here that the changed spectrum of microbial enzymes involved in post-translational modification of proteins (PTMP) may contribute to the aberrant modification of host proteins thus generating autoimmune responses by the host, resulting in autoimmune diseases.

## Introduction

Intricate host–microbe symbiotic relationships in the human gut have evolved during the long-term coevolution between the two. It resulted in fine-tuned inter kingdom molecular adaptations that benefit both sides (Donia and Fischbach, 2015). In particular, the commensal microbiota benefits from the continuous food supply, exogenous and endogenous, and constant physico-chemical conditions. Advantages for the host organism include metabolic, structural, protective, and other beneficial functions exerted by the commensal microbiota. The importance of commensal microbiota for the proper development and functioning of the host immune system is also well-recognized (Paun and Danska, 2015).

Our knowledge on the topic expands continuously. The complex microbiota of our gastrointestinal tract consists of at least 1000 bacterial species, the majority of which belong to the Firmicutes and Bacteroidetes phyla (Qin et al., 2010). The microbiota composition and function is very dynamic and multiple environmental factors affect its quantity, quality and functionality. Age, mode of delivery, infant feeding practices, the use of drugs (antibiotics), diet, industrial food additives, epidemiology, climate, eco-catastrophes, migrations, and many more are the influential factors (Lerner, 2011; Chassaing et al., 2015; Lerner and Matthias, 2015a,b,c).

It is not our intention to cover all the aforementioned factors that may affect host-microbe interaction. Here, we will provide an update on the dysbiotic situations, where the normal composition of the commensal is compromised, which may result in pathologies such as autoimmune diseases. More specifically, we will be focusing on the microbial enzymatic machinery that is involved in post-translational modification of proteins (PTMP). We hypothesize that the enzymes produced by the dysbiotic microbial community may process luminal proteins differently than that of the normal community. The abnormal PTMP may produce neo-epitopes that are autoimmunogenic and may induce systemic autoimmune responses resulting in autoimmune diseases.

## The role of intestinal dysbiosis in human autoimmune diseases

Presently, it is apparent that the microbiota and its products have a profound effect on the development and maintenance of the immune system. Germ-free animals, for example, have an impaired immune system that can be functionally restored after the inoculation of commensal bacteria (MacPherson et al., 2001, 2002; Mazmanian et al., 2005). The extent of dependency of the immune system on commensals may even suggest the commensalocentric view. At the same time, not all commensals are alike. The dysbiotic populations, with no identifiable pathogens, can still confer the susceptibility to immune-mediated diseases (Paun and Danska, 2015). Our focus here on autoimmune diseases and several mechanisms of the microbial involvement in the promotion of autoimmunity have been suggested (Chervonsky, 2013). The first one is the molecular mimicry, where microbial peptides are identical, or similar enough to self-peptides. Second, it could be bystander activation during infection, with the induction of costimulation and cytokine production by APCs, which, at the same time, may presents self-antigens. The third suggested mechanism is the “amplification of autoimmunity by cytokines” elicited by microbial activation of professional APCs and the innate lymphoid cells to produce proinflammatory cytokines by T cells. And the fourth suggestion is the involvement of the whole human intestinal system, including dysbiotic community, exogenous enzymes produced by the dysbiotic populations, and the corresponding PTMP activity that generates neo-epitopes.

The multiple animal models of human autoimmune diseases (AD) suggest the direct involvement of commensal microbiota in disease development. Under the germ-free conditions no disease is developing in the animal models of IBD, rheumatoid arthritis and multiple sclerosis, supporting the notion of “no bugs, no disease,” while in some others they are only attenuated (Wu and Wu, 2012). In some models of the human ADs, causality is strengthened by the reintroduction of specific microorganisms restoring the disease severity.

Some members of the gut microbiota have been linked to ADs. Changing a single bacterial species and/or the entire commensal community can alter the outcome of a specific AD due to the imbalance of pathological/protective immune responses (Wu and Wu, 2012). The summary of specific microbial species in relation to defined animal models of ADs and their functions, in relation to disease progression, is shown in Table 1.

**Table 1:** Autoimmune disease and associated dysbiotic characteristics^*^.

**Autoimmune disease**	**Dybiosic characteristics**	**Microbial species**	**Action**
Inflammatory bowel disease	Reduction of *Firmicutes+Bacteroides*, overgrowth of *Proteobacteria*	*Proteus mirabilis, Klebsiella pneumoniae*	Induce
		*Segmented filamentous bacteria*	Induce
		*B. fragilis*	Attenuate
		*B. thetaiotaomicron*	Attenuate
Rheumatoid arthritis	Increased diversity, Increase in *Porphyromonas, Prevotella, Leptotricha*, and *Lactobacillus* species	*Porphyromonas gingivalis, Prevotella nigrescens*,	Induce
		*Segmented filamentous bacteria*	Induce
		*Lactobacillus bifidus*,	Induce
		*Clostridium*	Induce
Type 1 diabetes	Decreased diversity, Increased *Bacteroidetes*, Decreased *Bifidobacteria*, and butyrate-producing bacteria	*Bacteroides* *Segmented filamentous bacteria*	Attenuate Attenuate
Celiac disease	Increased diversity	*Lachnoanaerobaculum, Prevotella, Actinomycetes*	Induce
		*Lachnoanaerobaculum umeaense*	Induce
Multiple sclerosis	Decreased *Clostridia* clusters XIVa and IV and *Bacteroidetes*	*Segmented filamentous bacteria*	Induce
		*Bacteroides fragilis, Lactobacillus paracasei, Lactobacillus plantarum*	Attenuate

* Adapted from references (Wu and Wu, 2012; Lerner and Matthias, 2015b; Paun and Danska, 2015).

## Enzymes from dysbiotic populations capable of PTMP

Endogenous and microbial enzymes have the capacity of intestinal enzymatic neo-antigen generation by PTMP. The corresponding modifications taking place in the intestine include peptides crosslinking, de/amination/deamidation, de/phosphorylation, a/deacetylation, de/tyrosination, de/glutamylation, de/glycylation, ubiquitination, palmitoylation, glycosylation, galactosylation, arginylation, methylation, citrullination, sumoylation, and carbamylation. For example, human endogenous intestinal enzymes and microbial transglutaminases (tTg, mTg) induce multiple neo-epitopes on the Tg-peptide docked complex or citrullination by peptidylarginine deiminase resulting in the formation of autoantibodies in celiac disease and rheumatoid arthritis, respectively. A similar phenomenon is observed in other ADs. Bacterial species, their corresponding enzymes capable of PTM of host proteins and potential involvement in disease are listed in Table 2.

**Table 2:** **Bacterial enzymes capable of post-translational modification of host proteins and potential involvement in disease**.

**Bacterium**	**PTMP**	**Relation to disease**
*Porphyromonas gingivalis*	Peptidylarginine deiminase	RA, SLE, Felty's syndrome, periodontal disease (Muller and Radic, 2015)
*Bacteroides fragilis*	Ubiquitination	Inflammatory bowel and autoimmune diseases (Patrick et al., 2011)
*Shigella flexneri*	Demyristoylation	Affects cellular growth, signal transduction, autophagasome maturation, and organelle function (Burnaevskiy et al., 2013)
*Shigella flexneri*	Deamidation	Inhibits acute inflammatory responses (Sanada et al., 2012)
*Shigella flexneri*	Protein kinase	Prevents phospho-IκBα degradation and NF-κB activation to establish infection (Kim et al., 2005)
Gram-negative bacteria	Kinases, phosphatases, phospholyases, and serine/threonine acetylases	Host phosphoproteome modulation to establish infection (Grishin et al., 2015)
*Escherichia coli, Salmonella, Shigella, Chlamydia, and Legionella*	Ligases and deubiquitinases	Modulation of host ubiquitin pathways to establish infection (Zhou and Zhu, 2015)
*Streptococcus pyogenes, Enterococcus faecalis, Listeria monocytogenes, Streptococcus pneumoniae, Pseudomonas aeruginosa, Capnocytophaga canimorsus, Treponema denticola*,	Glycosidases	Alteration of host glycobiome for immunomodulation, adherence, and nutrition to establish infection (Sjögren and Collin, 2014)
*Legionella, Chlamydia, Bacillus*	Histone methylases	Remodeling the host epigenetic machinery (Rolando et al., 2015)
Short chain fatty acids-producing commensals	Inhibition of histone deacetylases	Anti-proliferative and anti-inflammatory effects (Schilderink et al., 2013)
Bacteria at mucosal surfaces	Proteases	Generation of host damage-associated molecular patterns (Sofat et al., 2015)

## The role of post-translational modification in autoimmunity induction

Bacteria possess an amazing capacity for adaptation and survival strategies, including differential expression of transcriptome and proteome, variations in growth physiology, and in developmental behavior. PTMP contribute substantially to this adaptability and bacterial cell cycle regulation (Grageasse et al., 2015). On the other hand, the microbial PTMP has a paramount significance to the host. Their enzymatic apparatus is capable to transform naïve/self or non-self-peptides to autoimmunogenic ones. Several examples are worth mentioning.

A well-described PTMP is the citrullination, where an arginine residue in a protein is converted to a citrulline residue. The host enzyme is activated during apoptosis, autophagy, and NETosis processes, which are well-known as being implicated in autoimmunity (Valesini et al., 2015). In fact, some anti-citrullinated protein antibodies may serve as good diagnostic markers for autoimmune diseases such as rheumatoid arthritis (RA), systemic lupus erythematosus (SLE), and Felty's syndrome (Muller and Radic, 2015). The citrullinated proteins have also been detected in polymyositis and IBD. Some bacteria such as *Porphyromonas gingivalis* possess the corresponding enzymes, peptidylarginine deiminases, which may citrullinate human proteins (Wegner et al., 2010). The authors suggested that *P. gingivalis*-mediated citrullination of bacterial and host proteins may lead to the generation of antigens driving the autoimmune response in RA. Experimental verification of the specificity and activity of peptidylarginine deiminase from *P. gingivalis* revealed that it is primarily cell surface associated, heat stable, and display the optimal activity under alkaline conditions characteristic for the inflamed environment (Abdullah et al., 2013). Moreover, the enzyme has very broad substrate specificity and modifies arginine residues in all positions of all proteins tested. Thus, its presence in inflamed tissue may foster autoimmune reactions by altering host epitopes. In experimental models of periodontal disease and arthritis it has been found that peptidylarginine deiminase from *P. gingivalis* is a key contributor to the pathogenesis of these diseases (Gully et al., 2014). In human subjects, there is a strong association between the presence of *P. gingivalis* peptidylarginine deiminase and diseases such as RA and periodontitis (Laugisch et al., 2015; Shimada et al., 2015). In both diseases, the microbial enzyme convert arginine to a citrulline residue, thus creating a neo-antigen.

More recently, an additional PTMP activity, lysine acetylation, has been detected in *P. gingivalis* in addition to its citrullination capacity (Butler et al., 2015). A unique homolog of the eukaryotic ubiquitin has been found in the predominant bacterium of the human gut, *Bacteroides fragilis* (Patrick et al., 2011). In eukaryotes post-translational regulation by ubiquitination plays an important role in regulation of intracellular proteolysis and modification of protein function. The ubiquitination process is also central for immune surveillance and response to invading pathogens. Its presence in a predominant human gut bacterium may have important implications for our understanding of AD development.

One of the factors contributing to autoimmune progression is the cellular environment that may change PTMP (Cañas et al., 2015). PTMP of histones alter the chromatin architecture thus generating “open” and “closed” states, and these structural changes can modulate gene expression under specific conditions. While methylation and acetylation are the best-characterized histone PTMP, citrullination by the protein arginine deiminases represents another important player in this process (Slade et al., 2014). Juxtaposition of citrullinated histones with infectious pathogens and complement and immune complexes may compromise the tolerance to nuclear autoantigens and promote autoimmunity (Muller and Radic, 2015). Like a double-edged sword, histones can be post-translationally modified also by Tg cross-linking, a well-described PTMP (Lerner and Matthias, 2015c). Likewise, microbial transglutaminase, a member of the Tg family, is known for its pivotal function in bacterial survival (Lerner and Matthias, 2015c). Bacterial glyosidases are involved in PTMP and many key proteins of the immune system are glycosylated (Sjögren and Collin, 2014). The glycosylation sites of IgE, IgM, IgD, IgE, IgA, and IgG are functionally important, and they are responsible for the well-documented association between alterations of the serum glycome and autoimmunity. The altered glycan theory of autoimmunity has been recently suggested (Maverakis et al., 2015). It implies that each AD has a unique glycan signature characterized by the site-specific relative abundances of individual glycan structures on immune cells and extracellular proteins. This especially concerns the site-specific glycosylation patterns of different immunoglobulin classes and subclasses (Maverakis et al., 2015).

A well-characterized example of PTMP is the tTg in celiac disease that will be discussed in the next section.

## Where PTMP does takes place in the gut?

This topic still needs to be revealed in greater details, but there are some hints for at least two ADs. In celiac disease, the autoantigen is tTg, capable of deamidating, or transamidating gliadin (Reif and Lerner, 2004; Lerner et al., 2015a). This PTMP occurs below the epithelium, resulting in neo-epitopes of gliadin docked on the tTg, inducing anti-tTg, or anti neo-epitope tTg autoantibodies. These are the well-known serological markers of celiac disease (Lerner, 2014; Lerner et al., 2015b). More recently, a family member of tTg, the microbial Tg that is heavily used in the food industry, has been shown as a potent inducer of specific antibodies in celiac disease patients (Lerner and Matthias, 2015d). Interestingly, the same food additive has been suggested as a new environmental trigger and potential inducer of celiac disease (Lerner and Matthias, 2015a,c).

A number of PTMPs are relevant to IBD. These pathways include phosphorylation, neddylation hydroxylation, and cleavage of cytokine precursor forms by the inflammasome. The interplay of these rapid response mechanisms enables rapid adaptation to incoming inflammatory signals. Cytokine induced barrier breakdown allows for bacterial translocation to the basal aspect of intestinal epithelial cells. Bacterial antigens and endogenous danger signals are recognized by the adaptive and innate immune system, triggering a variety of reactions including apoptosis, increased cytokine release, loss of tight junctional proteins, and barrier breakdown (Ehrentraut and Colgan, 2012).

In rheumatoid arthritis, citrullination is a major post-translational modification of arginine, which converts naïve peptides into the immunogenic neo-epitopes. This PTMP constitutes the basis for the specific prediction of disease activity due to the production of anti-citrullinated protein antibodies (Lerner and Matthias, 2015b). It has been suggested that infectious agents that release toxins such as lipopolysaccharides at mucosal surfaces may trigger the inflammatory response with a potential to cause citrullination of various proteins such as fibronectin, fibrinogen, and collagen (Sofat et al., 2015).

Theoretically, it can be assumed that PTMP may take place in the lumen, on the intestinal, or buccal mucosal surfaces, in the interepithelial spaces, or below the epithelium. There are more questions, however, than definitive answers.

## The hypothesis

We hypothesize here that the PTM enzymes of dysbiotic gut microbiota behave like a Trojan horse. They are essential for the microbial growth and survival in the gut, but are detrimental to the human host. This enzymatic machinery is capable of PTMP, turning naïve peptides to immunogenic ones by generating, or exposing neo-epitopes, thus compromising tolerance and inducing autoimmunity. Peptides crosslinking, de/amination/deamidation, de/phosphorylation, a/deacetylation, de/tyrosination, de/glutamylation, de/glycylation, ubiquitination, palmitoylation, glycosylation, galactosylation, arginylation, methylation, citrullination, sumoylation and carbamylation are some examples for PTMP taking place in the intestine. The corresponding microbial enzymes that encounter the closely related host substrates under the optimal local conditions, act as post-translational modifiers of the host's peptides in the initiation, progression and maintenance of human systemic ADs.

## Summary

There are a number of factors, genetic and environmental, that have been identified as conducive to dysbiotic conditions in humans. We hypothesize here that the spectrum and activities of enzymes, which are normally involved in PTMP, become biased in the dysbiotic microbial community. The examples are given demonstrating how these activities may affect post-translational modification of host proteins thus generating new aberrant epitopes. These epitopes may generate host autoimmune responses and trigger autoimmune diseases. Much less is known, however, how to convert the “pathobiota” back to the “normobiota” to restore the balanced host-microbe interaction with a normal PTMP pattern. Answering this question will be the basis for the development of efficient therapeutic strategies to prevent autoimmune diseases.

## Author contributions

AL and RA designed, performed literature search and wrote the manuscript; TM designed, constructed table, edited and wrote the manuscript.

### Conflict of interest statement

The authors declare that the research was conducted in the absence of any commercial or financial relationships that could be construed as a potential conflict of interest.
